# Single Silver Nanoparticle Instillation Induced Early and Persisting Moderate Cortical Damage in Rat Kidneys

**DOI:** 10.3390/ijms18102115

**Published:** 2017-10-10

**Authors:** Elisa Roda, Sergio Barni, Aldo Milzani, Isabella Dalle-Donne, Graziano Colombo, Teresa Coccini

**Affiliations:** 1 Laboratory of Clinical & Experimental Toxicology and Poison Control Centre and National Toxicology Information Centre, Toxicology Unit, ICS Maugeri SpA—Benefit Corporation, IRCCS Pavia, via Maugeri 10, 27100 Pavia, Italy; elisa.roda@icsmaugeri.it; 2 Department of Biology and Biotechnology “L. Spallanzani”, Laboratory of Cell Biology and Neurobiology, University of Pavia, via Ferrata 9, 27100 Pavia, Italy; barni@unipv.it; 3 Department of Biosciences, Università degli Studi di Milano, via Celoria 26, 20133 Milano, Italy; aldo.milzani@unimi.it (A.M.); isabella.dalledonne@unimi.it (I.D.-D.); graziano.colombo@unimi.it (G.C.)

**Keywords:** nanotoxicity, in vivo, histology, renal ultrastructure

## Abstract

The potential toxic effects of silver nanoparticles (AgNPs), administered by a single intratracheal instillation (i.t), was assessed in a rat model using commercial physico-chemical characterized nanosilver. Histopathological changes, overall toxic response and oxidative stress (kidney and plasma protein carbonylation), paralleled by ultrastructural observations (TEM), were evaluated to examine renal responses 7 and 28 days after i.t. application of a low AgNP dose (50 µg/rat), compared to an equivalent dose of ionic silver (7 µg AgNO_3_/rat). The AgNPs caused moderate renal histopathological and ultrastructural alteration, in a region-specific manner, being the cortex the most affected area. Notably, the bulk AgNO_3_, caused similar adverse effects with a slightly more marked extent, also triggering apoptotic phenomena. Specifically, 7 days after exposure to both AgNPs and AgNO_3_, dilatation of the intercapillary and peripheral Bowman’s space was observed, together with glomerular shrinkage. At day 28, these effects still persisted after both treatments, accompanied by an additional injury involving the vascular component of the mesangium, with interstitial micro-hemorrhages. Neither AgNPs nor AgNO_3_ induced oxidative stress effects in kidneys and plasma, at either time point. The AgNP-induced moderate renal effects indicate that, despite their benefits, novel AgNPs employed in consumer products need exhaustive investigation to ensure public health safety.

## 1. Introduction

With the recent advances in nanotechnology, the increasing exploitation of engineered nanoparticles (NPs) has raised great concern regarding the potential for manufacturer and consumer exposure. Many of these manufactured nanomaterials are composed of metal and metal oxides, and several studies have evidenced the presence of metal-containing NPs in different occupational environments [1,2,3]. Metal NPs have achieved meaningful attention due to their technological interest, based on their excellent properties owing to their large surface area to volume ratio. Among these NPs, AgNPs have demonstrated an application in optical receptors, bio-labeling, and intercalation materials for electrical batteries. Furthermore the peculiar activities of silver nanoparticles have resulted in their widespread uses in consumer products and medical devices. Specific details for the current worldwide applications of AgNPs are summarized in reviews by McGillicuddy et al. [4] and Mackevica and Foss Hansen [5].

The risk of exposure to silver nanoparticles is ever increasing for both workers at nanosilver-manufacturing workplaces and consumers using nanosilver-containing products [6]. It is well known that NPs exposure, by direct or indirect mechanisms, may lead to unexpected distant responses, with overt toxic effects also involving distant organs e.g., kidneys [7,8]. The toxicity of nanosilver is still a highly debated topic in the literature and in the reports of several regulatory bodies. In fact, although the toxicology of silver and its compounds is well known, there are still gaps in the risk assessment of silver in the form of AgNPs [9,10,11].

Although a body of literature has been published on nanosilver, the main uncertain issue to be clarified regarding the nanosilver safety is to assess the toxic potential of nanosilver compared to that of its bulk counterpart material, also considering that the size, shape, and surface coatings of such AgNPs may greatly impact on their toxicity.

Several toxicity studies on AgNPs have been performed on bacteria and non-mammalian animal species, providing still comparatively limited data [12]. A number of experimental studies, both in vitro and in vivo, have pointed out toxic properties of nanosilver in a wide range of concentrations and sizes. Smaller AgNPs have been shown to be more active in exerting toxicological responses, with faster rates of Ag dissolution and higher cytotoxic potential at sub-acute time-points in vitro [13], also inducing organ toxicity and inflammatory responses after repeated oral administration in vivo [14]. Other investigations suggest the mild toxicity of AgNPs, which become toxic only when oxidized to silver ions, Ag+. Numerous in vitro studies have demonstrated the size-, dose- and cell-dependent cytotoxic and genotoxic effects of AgNPs, including reactive oxygen species (ROS) generation and lipid peroxidation, with the consequent production of carbonylated proteins (PCOs) [15]. Protein carbonyl formation is caused by the introduction into proteins of carbonyl derivatives such as aldehydes and ketones, generated from direct oxidation processes or from secondary protein reaction with reactive carbonyl compounds, eventually forming advanced glycation end products (AGEs) and advanced lipoxidation end products (ALEs) [16]. Moreover, aldehydes, as well as ROS, can affect a variety of biological processes, including transcription factor activation, gene expression, and production of inflammatory cytokines [17].

Several in vivo studies have been conducted using different routes of exposure, with the effort to depict AgNPs kinetics, tissue distribution and overall toxicity, also employing diverse animal species to evaluate different biological responses [12,14,18,19,20]. AgNP toxicological data coming from in vivo studies pointed out (i) diverse toxic responses on different target organs and systems, either after single or repeated administration and different treatment routes [12,21,22,23,24,25]. In particular, several investigations (see review by Iavicoli et al. [26]) proved that AgNPs are able to induce serious functional and structural alterations in all kidney regions (i.e., cortex, inner and outer medulla), causing for example glomerular and Bowman’s space modification, swelling and cytoplasmic vacuolization of the tubular epithelium, basement membrane thickness, and swollen podocytic secondary processes. Nevertheless, it should be underlined that these results need to be interpreted with care since high and unrealistic single exposure doses and non-physiological routes of exposure have been used.

In the current study we adopted an integrated approach including both in vivo toxicology studies and physicochemical characterization of the AgNPs, to assess the potential toxic effects associated with the exposure to a commercial nanosilver. Histochemical and molecular endpoints, in terms of histopathological changes, overall toxic response and oxidative stress (i.e., tissue/organ and plasma protein carbonylation), paralleled by ultrastructural observations using transmission electron microscopy (TEM), were carried out to examine renal responses in rats after intratracheal instillation (i.t.) of a low dose of AgNPs (50 µg/rat).

The effects of AgNPs was assessed at 7 and 28 days post-exposure in comparison with those caused by administration of an equivalent dose of ionic silver in the form of silver nitrate (7 µg AgNO_3_/rat, corresponding to 4.4 µg Ag).

## 2. Results

No significant toxicity signs or mortality were observed in the animals after exposure to both AgNP or AgNO_3_, neither 7 days post treatment nor during the recovery periods (until 28 days). The body weight (b.w.) gain of treated rats was comparable to that measured from controls: the b.w. from day zero to the last day of treatment (day 28) increased by about 40% in all groups.

### 2.1. Model Silver Nanoparticles (AgNPs): Physicochemical Characteristics

**Figure 1:**
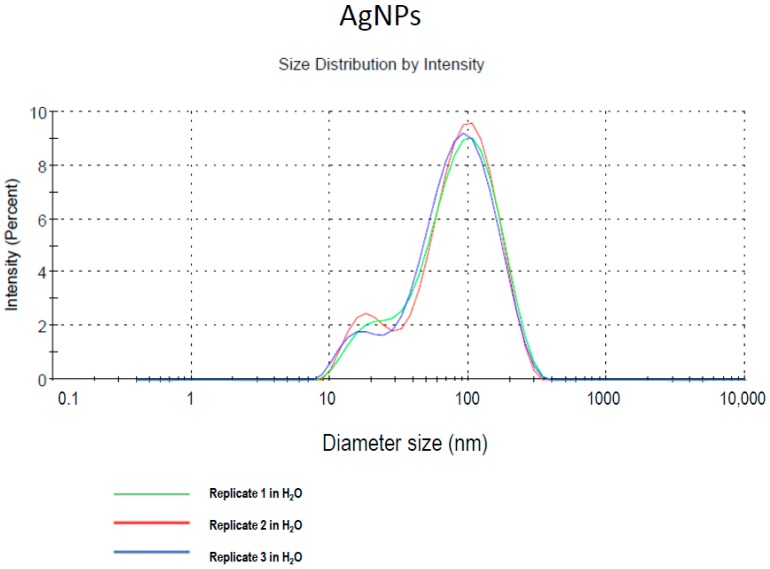
Size distribution determined by dynamic light scattering (DLS) measurements of model AgNPs in deionized water. Modified from Coccini et al. [27].

The characteristics of AgNP 1% in water, with the appearance of brown color were 1 g/cm^3^ density, 3 mPa/s viscosity (25 °C), <0.50 PdI, 6.5 pH, and 20 nm nominal hydrodynamic size diameter. Dynamic light scattering determination (Malvern Instruments Ltd., Malvern, UK) of AgNP size distribution shows two populations of particles in the ranges of 20 and 100 nm, respectively (Figure 1). Particle samples were vortexed prior to measurement although no tendency to agglomerate was detected. For an exhaustive physicochemical characterization, see [27].

### 2.2. Histopathology: Haematoxylin & Eosin (H & E) Staining

**Figure 2:**
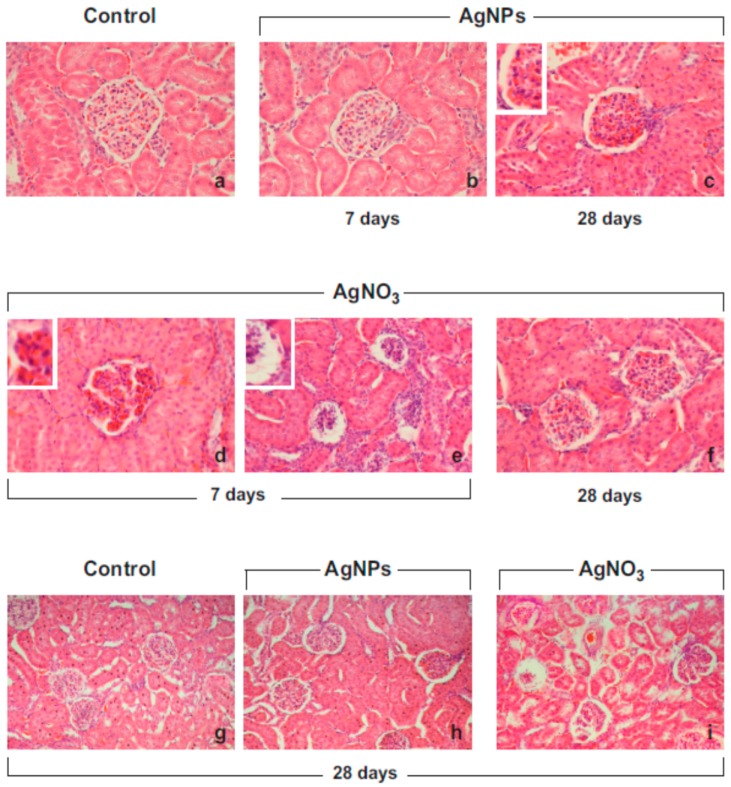
Representative H&E-stained cortical renal specimens from control animals (**a**,**g**) and rats exposed to AgNPs (**b**,**c**,**h**) or AgNO_3_ (**d**–**f**,**i**), 7 and 28 days post-instillation. The physiological renal structure is clearly well preserved in control rats (**a**,**g**), while moderate morphological alterations are manifest in the renal cortex of the treated animals, at both evaluated time points. Seven days post-i.t., the dilatation of the intercapillary and peripheral Bowman’s space was observed (**b**,**d**,**e**), often accompanied by glomerular shrinkage (**d**); these effects were still evident after both treatments at day 28 (**c**,**h** for AgNPs and f, **i** for AgNO_3_) accompanied by the appearance of several glomerular areas of interstitial micro-hemorrhages. Objective magnification: 40× (**a**–**d**,**f**); 20× (**e**), 10× (**g**–**i**).

Light microscopy photomicrographs of H & E-stained tissues showed histopathological changes of the renal architecture mainly localized in the cortical area, already observable 7 days after exposure, and lasting until day 28, to both AgNP and AgNO_3_ materials, (Figure 2) with a slightly more marked effect caused by AgNO_3_ (Table 1).

Seven days after both AgNO_3_ or AgNPs instillation, the main alterations were the enlargement of the Bowman’s capsule paralleled by glomerular shrinkage. Specifically, in some places glomeruli appeared deformed, often collapsed or showing packed mesangial and endothelial cells (Figure 2). Edematous, hemorrhagic glomeruli were found 28 days after both treatments, displaying the alterations of the vascular component of the renal mesangium, with the capillary loops sometimes distorted, and evident interstitial micro-hemorrhages (Figure 2). Within the cortical area, the AgNP- and AgNO_3_-dependent effects were mainly depicted at the glomerular level, while no evident alterations were observed at tubular level, with AgNO_3_ inducing a more marked effect.

### 2.3. Transmission Electron Microscopy (TEM) Observations: UA & LC Staining

**Figure 3:**
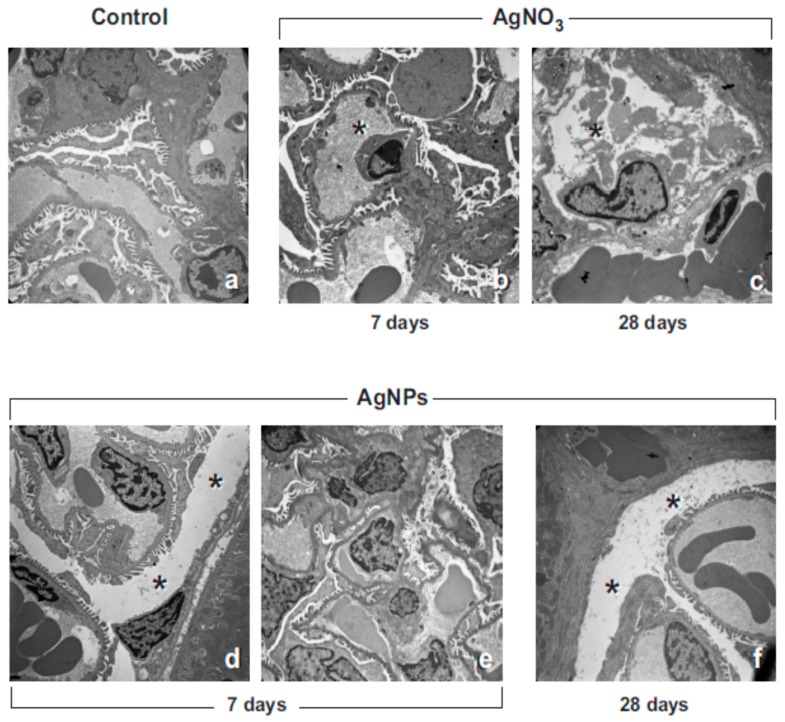
Transmission electron microscopy images (UA & LC staining) showing moderate renal cortical alteration 7 and 28 days after AgNP- and AgNO_3_-instillation. The evident dilatation (asterisks) of the intercapillary and peripheral Bowman’s space (**d**,**f**) is shown together with shrinkage of both mesangial cells and glomerular capillaries; endothelial and podocytic cells appeared damaged (**b**,**c**, asterisks), the latter showing the podocyte foot processes scarcely developed (**e**). Occasional expression of cell injury, namely apoptotic cell death and cytoplasm segmentation are also presented (**b**,**c**). Original magnification: ×4400 (**a**–**c**); ×3000 (**d**–**f**).

The renal cortical tissues of the control rats revealed a normal physiological appearance of the kidney’s (i.e., corpuscles and tubules) ultrastructural patterns (Figure 3a). In contrast, in accordance with histopathological evidence, ultrastructural changes as a consequence of AgNP and AgNO_3_-exposure were demonstrated (Figure 3b–f and Table 1). Specifically, after AgNP exposure, the renal corpuscles displayed extensive congestion, filling up the glomerular capillary loops. Some glomeruli were shrunk with concurrent dilatation of the intercapillary and peripheral Bowman’s space (Figure 3d); nevertheless, the glomerular basement membrane displayed a normal physiological thickness. Shrinkage of both mesangial cells and glomerular capillaries was also observed, accompanied by the presence of scarcely-developed podocyte foot processes (Figure 3e). The same changes were observed in the cortical area of AgNO_3_-exposed rats, where cell death was also revealed (Figure 3b,c). Specifically, no presence of necrosis was detected, nonetheless paralleled by the presence of several apoptotic cells; damaged endothelial and podocyte cells, ultra-structurally characterized by nuclear pyknosis, karyorrhexis, cytoplasmic segmentation and apoptotic body formation, were found after AgNO_3_ treatment (Figure 3b,c), already at the early time point (day 7) and persisting until day 28.

### 2.4. Oxidative Stress Evaluation by Protein Carbonylation Assessment in Kidney Slice Homogenates and Plasma

**Figure 4:**
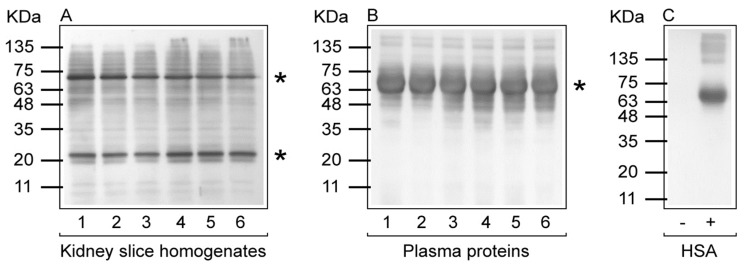
Protein carbonylation in rat kidney slice homogenates and plasma proteins. Representative Western blot with anti-DNP antibody for PCOs of kidney slice homogenates (**A**) and plasma proteins (**B**) of adult male Sprague–Dawley rats treated with a single i.t. instillation of AgNPs or AgNO_3_. In both (**A**) and (**B**): 1, control (7 days); 2, AgNO_3_ (7 days); 3, AgNPs (7 days); 4, control 28 days); 5, AgNO_3_ (28 days); 6, AgNPs (28 days). (**C**) human serum albumin (HSA) in the reduced form (−) and in the carbonylated/oxidized form (+). Protein molecular weight markers are shown on the left side of each panel. Although there were no differences between controls and treated samples, the most carbonylated proteins are highlighted by an asterisk (*) in kidney and plasma samples.

PCOs are widely analyzed as a measure of severe protein oxidation and employed as a biomarker of oxidative stress. One of the most commonly used method for determining PCOs relies on the derivatization of the carbonyl group with 2,4-dinitrophenylhydrazine (DNPH), which generates the stable 2,4-dinitrophenylhydrazone (DNP)-carbonyl adduct. Derivatized PCOs can be detected quantitatively by immunoassay using high specificity antibodies against DNP. The results of protein carbonylation assessed by Western blotting using anti-DNP antibodies from rats treated with AgNPs or AgNO_3_ are shown in Figure 4. At both considered time points (i.e., 7 and 28 days after a single i.t. instillation), treatment with AgNPs or AgNO_3_ did not induce any increase in protein carbonyls compared with controls, in both kidney homogenates and plasma proteins. No immunostaining was observed in parallel experiments in which either the protein samples were treated with NaBH_4_ or the primary antibody to DNP was omitted.

## 3. Discussion

In the latest decades, growing worldwide attention has been devoted to engineered NPs based on their putative novel applications in a variety of consumer and occupational fields.

However, not enough studies have ruled out the potential hazards due to NPs and hence the definitive conclusions and tools, technologies, systems, and methods to obviate the risks are still elusive. Several tools for the assessment of the risks are still at the conceptual stage, and further, there are considerable uncertainties as to how to measure NP exposure (i.e., which metrics to adopt systematically) and to assess their toxicity [28].

Thus, being unable to perform a quantitative risk assessment for NPs, it should be made mandatory to prevent exposure by appropriate precautionary measures and practicing best industrial hygiene to avoid future shock scenarios from environmental or occupational exposures [29,30].

In the safety assessment area, it is crucial to define adequate strategies and establish whether NP tailored testing methods should be added to conventional toxicity testing protocols to comply with regulatory demand and properly characterize NP potential hazards.

According to the major institutions and international consensus meetings, a proposed multi-tiered testing protocol is recommended to address toxicological research and health risk assessment for NPs. The toxicity strategy should firstly include an exhaustive physicochemical characterization, the use of validated cellular (in vitro) models, followed by limited and focused in vivo studies. Specifically, investigations in laboratory animals may provide essential insight, providing pivotal data, contributing to clarifying the NP toxicity mechanisms, also allowing us to understand toxicity targets at different molecular levels, to improve the overall knowledge of the adverse outcomes that may result from NP exposure [29,30,31].

The present in vivo investigation addressed the renal effects of AgNPs compared to a bulk counterpart material i.e., AgNO_3_, investigating histochemical and molecular endpoints, 7 and 28 days after a single i.t. instillation.

Our data demonstrated that a single AgNP i.t. low dose administration caused moderate renal damage, in term of histopathological and ultrastructural alteration, in a region-specific manner, with the cortex the most affected area, without generation of PCOs. Notably, the employed counterpart silver nitrate (AgNO_3_) caused similar adverse effects with a slightly more marked extent, specifically also triggering apoptotic phenomena.

Specifically, (i) 7 days after exposure to both AgNPs and AgNO_3_, the main renal changes concerned the Bowman’s capsule (in terms of dilatation of the intercapillary and peripheral Bowman’s space) as well as the glomerular shrinkage; (ii) after 28 days these effects still persisted after both treatments, accompanied by a further injury involving the vascular component of the renal mesangium, with evident interstitial micro-hemorrhages. These overall changes were slightly more marked after AgNO_3_ exposure compared to AgNP, with apoptotic phenomena observed in AgNO_3_-treated animals only.

The evaluation of PCOs in both kidney (organ response to oxidative stress) and plasma (systemic response to oxidative stress) samples indicated that a single exposure to AgNPs or AgNO_3_ was devoid of any oxidative stress effects, at both the considered time points (i.e., 7 and 28 days after treatment), as demonstrated by PCO occurrence similar to that observed in control rats.

A bulk of experimental investigations indicate that, despite the indubitable AgNP benefits, they may pose a serious hazard to various organisms [21,32,33,34,35,36]. Hence, there is a need for research on low-level toxicity of nanosilver, which may mimic the occupational, environmental and consumer exposure scenario [37,38]. To this aim, the present study tested a dose of 50 µg AgNPs/rat, corresponding to a dose of about 200 µg/kg b.w., based on a previous investigation by Takenaka et al. [37], reporting lung toxicity as well as NP translocation to the systemic circulation with consequent deposition in distant organs, e.g., kidney, after a single i.t. exposure to AgNPs with 15 nm modal diameter. A parallel study by the same authors, using inhalation as the route of treatment, employing the identical NP type (AgNPs of about 15 nm), at a correspondent dose of 133 μg/m^3^ AgNPs (for 6 h exposure), showed that the highest concentrations of silver were found in the lungs and blood, whereas low concentrations were measured in the liver, kidneys, spleen, brain and heart, as a consequence of translocation, i.e., the ability to reach secondary organs via the bloodstream [37].

Another recent work by Silva et al. [38] tested a range of low AgNP doses (i.e., from 30 to 300 µg/rat) with different nominal diameters (i.e., 20 and 110 nm) using a single i.t. exposure. The authors established that these selected doses would approximate human occupational and consumer exposure in a worst-case scenario. All AgNP types produced significant inflammation throughout the experimental time course (1–21 days), after 150 and/or 300 µg/rat doses. Pulmonary histology alterations were also detected in animals exposed to 300 µg/rat AgNPs only.

Moreover, it has to be mentioned that Takenaka and colleagues [37] demonstrated by inductively coupled plasma mass spectrometry (ICP-MS) analysis that only 9–16 μg of the 50 μg AgNP instilled was retained in the lung on day one after the i.t. exposure, remaining almost unchanged until day 7. In contrast, rapid clearance of instilled water-soluble AgNO_3_ from the lung was observed. When AgNPs were tested by inhalation, Ag content was also determined in different organ tissues. In particular, Ag content in the kidney was 45 ± 10 ng one day after exposure, decreasing to approximately 5 ± 5 ng at day four.

In line with these data, another recent investigation [39] testing AgNPs at a total dose of 100 µg/mouse administered by oropharyngeal aspiration (20 µg single dose/aspiration, once a week for five weeks), demonstrated pronounced pulmonary toxic effects paralleled by NP translocation to secondary organs, determined two and 28 days after final treatment. Specifically, renal Ag content, measured by ICP-MS, was about 0.01 ng/mg tissue at the first evaluated timepoint, decreasing about three- to four-fold at the latest detection.

In a recent study by Arai et al. [40], 20 nm AgNP suspension and AgNO_3_ solution were i.t. instilled at a dose of 10 µg Ag/mouse. Pulmonary toxicity in terms of bronchoalveolar lavage (BALF) alterations (e.g., an increase of proinflammatory cytokines and neutrophils), as well as translocation to distant organs, were demonstrated after exposure to both compounds. The concentration of Ag in the lung tissue of AgNP-treated mice was significantly higher than that in AgNO_3_-treated mice at four and 24 h after instillation. Furthermore, in AgNP-treated mice, only a trace amount of Ag was detected in the liver, while, in contrast, 7% of the initial dose of Ag was recovered in the liver of AgNO_3_-treated mice 4 h after treatment, suggesting that the ionic form of silver was absorbed by the lung tissue and entered the systemic circulation more efficiently than AgNPs. In fact, the renal Ag concentration in AgNO_3_-instilled mice was 0.046 ± 0.033 µg/g tissue, corresponding to about 2% of the initial Ag instilled dose. Differently, in AgNP-treated animals, the Ag concentration in the kidney was undetectable, being below Limit of detection (LOD) at both considered timepoints, i.e., 4 and 24 h after i.t. administration.

In line with the above literature, in the present study a low dose of AgNPs was tested compared to an equivalent dose of ionic silver in the form of AgNO_3_. The chosen dose (50 µg/rat corresponding to 200 µg/kg b.w.) and the AgNP type were used to mimic occupational and consumer exposure to engineered NPs (ENPs) used in paints and coatings, in that, among the industrial applications, coatings, paints, and pigments are the most important applications of ENPs in terms of overall use [39,41].

Occupational exposure to AgNPs used in paints and coatings can occur during the production process, handling and application. Activities such as scratching (by children, pets), cleaning, UV exposure, and demolition can damage the coatings resulting in the release of NPs into the environment. Respiratory inhalation is the most probable route of exposure to environmental dust [42]. Several investigations have further demonstrated that NPs entering the body through the pulmonary system or another portal of entry reached the blood circulation and were then transported to various distant target organs [43,44,45,46,47].

According to the well-known NP translocation ability, our results contribute to pointing out the modest involvement of a secondary organ (i.e., the kidney, a known target organ for silver toxicity) in the action of AgNPs introduced by pulmonary instillation. The moderate renal response may be due to the migration of the nanoparticles from the lung to the systemic circulation or to secondary organ changes caused by circulating inflammatory factors released from the lung following local insult [48]. A direct renal action of silver ions released from the absorbed AgNPs can also not be excluded [47], although good stability of the silver NPs should be postulated based on a recent study using an in silico PBPK (physiologically based pharmacokinetic) model by Bachler and colleagues [49], which demonstrated that AgNPs administered in vivo are directly stored as insoluble salt particles rather than dissolved into Ag+.

In the current investigation, the kinetics of Ag distribution was not investigated based on previous experimental works, employing similar doses and demonstrating renal Ag content below the detection limit 24 h after exposure [40].

According to previous data, revealing AgNP accumulation in the kidney cortical and medullar regions, mainly deposited in the top of the cortical glomeruli within the cytoplasm of mesangial cells [19,50,51,52], our current investigation evidenced moderate renal alterations, in terms of the enlargement of the Bowman’s capsule paralleled by glomerular shrinkage 7 days after AgNP i.t. low dose administration, with a persistence of these effects at day 28, with a further injury involving the vascular component of the renal mesangium. Morphological and ultrastructural renal modification could be paralleled by functional consequences, such as tubular dysfunction or glomerular filtration damage. In this view, the use of some functional parameters could allow the detection of AgNP-induced early renal dysfunction. In particular, as previously demonstrated by Fontana and colleagues [53] using metal NPs, potential peripheral markers of renal NP-induced effects, such as urinary retinol binding protein and β2-microglobulin, could be extremely useful for risk assessment and management in occupational and environmental exposure scenarios.

In conclusion, our data demonstrated that even following a single i.t. instillation of a low dose, AgNPs may moderately affect renal tissue, indicating that, despite the incontestable benefits, novel AgNPs employed in a huge number of consumer products may pose a threat to public health. This concern implies a careful investigation approach consisting of detailed toxicological studies paralleled by a comprehensive physico-chemical characterization.

## 4. Materials and Methods

### 4.1. Chemicals

The AgNPs were kindly provided by Colorobbia (Colorobbia S.p.A., Vinci, Italy), series PARNASOS NAMA 39 1103 F01 1%, for details see Section 2.1. Haematoxylin and Eosin alcoholic solution staining were purchased from Ettore Pasquali S.r.L. (Milan, Italy). The 2,4-dinitrophenylhydrazine (DNPH) was purchased from Sigma-Aldrich (Sigma-Aldrich, Milan, Italy). Anti-dinitrophenyl-KLH (anti-DNP) antibodies, rabbit IgG fraction and goat anti-rabbit IgG, horseradish peroxidase conjugate, were purchased from Molecular Probes (Eugene, OR, USA). ECL Plus Western blotting detection reagents were obtained from GE Healthcare (Milan, Italy). All reagents of analytical grade were kindly provided by the Eureka Lab Division (Chiaravalle, AN, Italy). All other reagents were purchased from Sigma-Aldrich (Milan, Italy).

### 4.2. Animals and Experimental Design of Silver Exposure

Adult male Sprague–Dawley rats (12 weeks old), purchased from Charles River Italia (Calco, Italy), were allowed to acclimatize for at least two weeks before treatment, and kept in an artificial 12 h light:12 h dark cycle with humidity at 50 ± 10% throughout the experiment. Animals were provided rat chow (VRF1 Mucedola diet) and tap water ad libitum.

The weight of the rats in all treatment groups at time point zero (day of instillation) was 253 ± 9 g. Groups (*n* = 6 total for each treatment group at each time point) of rats were treated with a single intratracheal (i.t.) instillation of AgNPs (50 µg/rat). Separate groups of animals received an equivalent i.t. dose of ionic silver in the form of AgNO_3_ (7 µg/rat, corresponding to 4.4 µg Ag) or 0.1 mL saline/rat (as control). Appropriate doses, durations and routes of exposure were chosen accordingly to a previous study by Takenaka et al. [37], showing that a single i.t. dose of 50 µg AgNPs/rat induced lung toxicity and NP translocation to the target organ i.e., kidney. Although intratracheal instillation (i.t.) is a more artificial route of dosing bolus material to the lungs and there are differences in the distribution, behavior, clearance and retention of materials when administered by i.t. compared to inhalation, the former is widely used to address specific endpoints regarding the toxicity of nanomaterials [37,40,54,55,56,57,58].

The AgNP suspension was vortexed on ice just before exposure to force nanoparticle dispersion and avoid the formation of agglomerates. No surfactants or solvents were used. 7 and 28 days after treatment, the rats were euthanized with an overdose i.p. injection of 35% chloral hydrate (100 mL/100 g body weight (b.w.)). Blood was withdrawn and centrifuged to obtain plasma, and the kidneys were excised. All samples were processed for (i) histopathology (i.e., H & E staining), (ii) ultrastructural evaluation by transmission electron microscopy (TEM) and (iii) oxidative stress evaluation by protein carbonylation determination. Specifically, protein carbonylation is a major form of protein oxidation and is widely used as an indicator of oxidative stress [59,60,61].

The study was conducted within the CARIPLO Project—Rif. 2011–2096, under Italian Legislation of research protocol, in compliance with the European Council Directive 2010/63/EU on the care and use of laboratory animals. A research protocol entitled “Molecular and cellular studies on metal-doped nanoparticles. A research strategy aimed at characterizing the biological risk”, was submitted (delivery notice n. 13898498944-4, 1 April 2010) to the Italian Ministry of Health which formally approved it with a tacit consent. All animals used in this research were treated humanely according to institutional guidelines, with due consideration for the alleviation of distress and discomfort. Rats were anesthetized with pentobarbital sodium before i.t. instillation with the test materials.

### 4.3. Kidney Histology and Ultrastructural Morphology Evaluations

For each treatment at each time point, renal tissues were processed for the following morphological evaluations.

#### 4.3.1. Rat Renal Specimen Preparation and Histology

At necropsy, the kidneys from control and treated animals were carefully removed and hemi-dissected. One half of each kidney was washed in NaCl 0.9% and post-fixed by immersion for 7 h in 4% paraformaldehyde in 0.1 M phosphate buffer (pH 7.4), dehydrated through a graded series of ethanol and finally embedded in Paraplast. Seven micrometer-thick sections of the samples were cut in the coronal plane and collected on silane-coated slides. Subsequently, to evaluate overall structural changes by light microscopy, H & E staining was performed. The slides were then observed and scored with a bright-field Zeiss Axioscop Plus microscope (Carl Zeiss S.p.A., Milan, Italy). Specifically, five slides (20 sections) per animal (*n* = 6) were analyzed; five microscopic fields were examined in each section for each rat per time/condition.

The images were recorded with an Olympus Camedia C-5050 digital camera and stored on a PC running Olympus software (Olympus Italia S.r.l., Segrate, Italy).

#### 4.3.2. Transmission Electron Microscopy (TEM): UA & LC Staining

Kidney fragments (small blocks of about 1 mm^3^) were fixed for 4 h by immersion in ice-cold 1.5% glutaraldehyde (Polysciences, Inc., Warrington, PA, USA) buffered with 0.07 M cacodylate buffer (pH 7.4), containing 7% sucrose, followed by post-fixation in OsO_4_ (Sigma Chemical Co., St. Louis, MO, USA) in 0.1 M cacodylate buffer (pH 7.4) for 2 h at 4 °C, dehydrated in a graded series of ethanol and embedded in Epon 812. For light microscopy pre-examination, semithin sections (1 micrometre thick) were stained with 1% borated methylene blue. For electron microscopy, ultrathin sections (about 600 Å thick) were cut from the blocks, mounted on uncoated 200-mesh-copper grids, and doubly stained with saturated uranyl acetate in 50% acetone and Reynold’s lead citrate solution. The specimens were examined with a Zeiss EM 300 electron microscope (Carl Zeiss S.p.A) operating at 80 kV.

#### 4.3.3. Semiquantitative Kidney Lesion Analysis

A scoring system was used to evaluate the extent of renal damage using conventional brightfield microscopy according to a semiquantitative scale ranging from undetectable (−) to severe (+++). Specifically, 20 sections per *n* = 3 animals were analyzed, examining five microscopic fields in each section for each rat per time/condition.

The localization and degree of lesions was recorded and graded as follows: (−) absent/undetectable lesions; (+) mild injury; (++) moderate damage; (+++) severe alteration. Specifically, the following alterations were recorded: Bowman’s capsule enlargement, mesangial cells and glomerular capillaries shrinkage, edema and interstitial micro-hemorrhages, podocytic foot process alteration, apoptotic phenomena.

#### 4.3.4. Statistical Analysis

Differential renal lesion extent data were not normally distributed; therefore, the Kruskal–Wallis nonparametric test was used. Statistical significance is indicated with a * (*p* value < 0.05).

### 4.4. Oxidative Stress Evaluation: Detection of Protein Carbonylation in Kidneys and Plasma

Control, AgNO_3_ and NP-treated (7 and 28 days) kidney slices were homogenized with potter (40 cycles) in ice-cold lysis buffer (50 mM Tris–HCl, pH 7.4, 150 mM NaCl, 5 mM EDTA, 1% Triton X-100 supplemented with proteases inhibitors (P8340 SIGMA)). Lysates were incubated on ice for 30 min and centrifuged at 14,000× *g* for 15 min at 4 °C to remove tissue debris. Clarified supernatants were quantified for protein content using BCA assay and stored at −80 °C until protein carbonylation immunoassay. Plasma from control, AgNO_3_ and NPs-treated (7 and 28 days) mice were diluted 1:40 in 50 mM PBS pH 7.4, quantified for protein content using Bradford assay and stored at −80 °C until protein carbonylation immunoassay.

Kidney (10 µg) and plasma proteins (5 µg) were fractionated on 12% (*w*/*v*) reducing SDS-PAGE gels and electroblotted onto a polyvinylidene difluoride (PVDF) membrane. Protein carbonylation was detected, after derivatization with DNPH, with anti-DNP antibodies specific for the 2,4-dinitrophenyl hydrazone–protein adduct by Western blot immunoassay [59,60,61]. In particular, PVDF membranes were subsequently incubated in 2 M HCl and DNPH (0.1 mg/mL in 2 M HCl) for 5 min each. Membranes were then washed three times in 2 M HCl and seven times in 100% methanol for 5 min each, followed by one wash in PBST (10 mM Naphosphate, pH 7.2, 0.9% (*w*/*v*) NaCl, 0.1% (*vol*/*vol*) Tween-20) and blocking for 1 h in 5% (*w/v*) nonfat dry milk in PBST. After washing three times with PBST for 5 min each, carbonyl formation was probed by 2-h incubation with 5% milk/PBST containing anti-dinitrophenyl-KLH (anti-DNP) antibodies (1:10,000 dilution). After three washes with PBST for 5 min each, the membrane was incubated with a 1:40,000 dilution of the secondary antibody linked to horseradish peroxidase in 5% milk/PBST for 1 h. After washing three times with PBST for 5 min each, immunostained protein bands were visualized with enhanced chemiluminescence detection. Protein bands on PVDF membranes were then visualized by washing the blots extensively in PBS and then staining with amido black.

## Figures and Tables

**Table 1:** Semi-quantitative scoring of renal lesions in control and treated rats.

**7 Days**				***p* Value**
	**Control**	**AgNPs**	**AgNO_3_**	
Bowman’s capsule enlargement	−	++	+++	*
Mesangial cells and glomerular capillaries shrinkage	−	++	++	*
Edema and interstitial micro-hemorrhages	−	++	+++	*
Podocyte foot processes alteration	−	+	+	*ns*
Apoptosis	−	−	++	*
**28 Days**				***p* Value**
	**Control**	**AgNPs**	**AgNO_3_**	
Bowman’s capsule enlargement	−	++	+++	*
Mesangial cells and glomerular capillaries shrinkage	−	++	++	*
Edema and interstitial micro-hemorrhages	−	++	+++	*
Podocyte foot processes alteration	−	+	+	*ns*
Apoptosis	−	−	++	*

Degree scale: from absent (−) to severe (+++). *p* values calculated by Kruskal–Wallis test: (*) <0.05; *ns* = not statistically significant.

## Abbreviations

AgNPs	Silver nanoparticles
AgNO_3_	Silver nitrate
i.t.	Intratracheal instillation
H&E	Haematoxylin/Eosin staining
TEM	Transmission electron microscopy
DLS	Dynamic light scattering
AGEs	Advanced glycation end products
ALEs	Advanced lipoxidation end products
DNP	2,4-Dinitrophenylhydrazone
DNPH	2,4-Dinitrophenylhydrazine
PCOs	Protein carbonyls
PVDF	Polyvinylidene fluoride membrane
ROS	Reactive oxygen species
